# Synthesis and Acetylcholinesterase Inhibitory Evaluation of 4-(1,3-Dioxoisoindolin-2-yl)-*N*-Phenyl Benzamide Derivatives as Potential Anti-Alzheimer Agents

**Published:** 2016

**Authors:** Ahmad Mohammadi-Farani, Samira Soltani Darbandi, Alireza Aliabadi

**Affiliations:** a *PharmaceuticalSciencesResearchCenter, FacultyofPharmacy, KermanshahUniversityofMedicalSciences, Kermanshah, Iran. *; b *DepartmentofPharmacology, ToxicologyandMedicalServices, FacultyofPharmacy, KermanshahUniversityofMedicalSciences, Kermanshah, Iran*; c *.StudentsResearchCommittee, KermanshahUniversityofMedicalSciences, Kermanshah, Iran.*; d *DepartmentofMedicinalChemistry, FacultyofPharmacy, KermanshahUniversityofMedicalSciences, Kermanshah, Iran.*

**Keywords:** Synthesis, Phthalimide, Acetylcholinesterase, Alzheimer

## Abstract

Alzheimer᾽s disease is characterized by cognitive deficits, impaired long-term potentiation of learning and memory. A progressive reduction in cholinergic neurons in some areas of the brain such as cortex and hippocampus is related to the deficits in memory and cognitive function in Alzheimer’s disease (AD). In the current project a new series of phthalimide derivatives were synthesized. Phthalic anhydride was reacted with 4-aminobenzoic acid in the presence of triethylamine under reflux condition. Then, the obtained acidic derivative was utilized for preparation of final compounds via an amidation reaction through a carbodiimde coupling reaction. Anti-acetylcholinesterase activity of synthesized derivatives was assessed by Ellman᾽s test. Compound 4g in this series exhibited the highest inhibitory potency (IC_50_ = 1.1 ± 0.25 µM) compared to donepezil (IC_50_ = 0.41 ± 0.12 µM) as reference drug.

## Introduction

**Figure 1:**
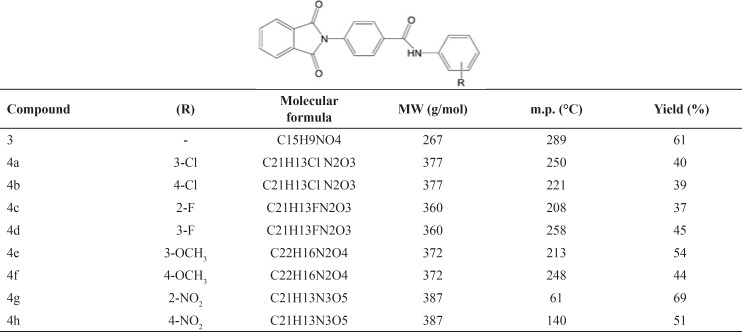
Structures of acetycholinesterase inhibitors in the market

Alzheimer᾽s disease, one of the most common neuron-degenerative diseases, is characterized by the appearance of senile plaques mainly composed of amyloid β (Aβ), and by the development of neurofibrillary tangles in patients′ brain. Patients with Alzheimer᾽s disease have cognitive deficits, impaired long-term potentiation of learning and memory (1). A progressive reduction in cholinergic neurons in some areas of the brain such as cortex and hippocampus is related to the deficits in memory and cognitive function in Alzheimer’s disease (AD) (2-6). Alzheimer’s disease (AD) is the most prevalent cause of dementia with ageing. Pharmacological treatment of AD is based on the use of acetylcholinesterase inhibitors, which have beneficial effects on cognitive, functional, and behavioural symptoms of the disease. AChE inhibitors are the most widely developed compounds for the symptomatic treatment of this disease. Rivastigmine, tacrine, galantamine and donepezil are the current medications in the market prescribed for AD patients (Figure 1.).

**Figure 2:**
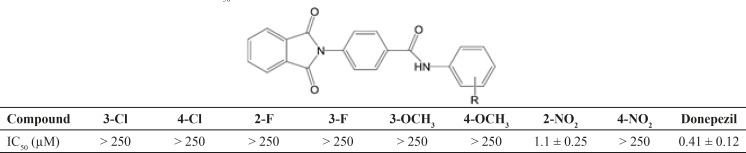
Structures of three phthalimide based anticholinesterase

According to the literatures, phthalimide derivatives have potential activity for inhibition of acetylcholinesterase enzyme (Figure 2.) (7-12). In fact, phthalimide-based compounds are capable of strong binding to the peripheral binding site (PAS) of the acetylcholinesterase.

However, in the current investigation, we focused on the design and synthesis of new anti-acetylcholinesterase analogs containing phthalimide substructure. In fact, phthalimide based compounds have similar pharmacophoric portions like indanone ring of the donepezil and are able to act as peripheral binding site inhibitor of AChE (13, 14). 

**Scheme 1:**
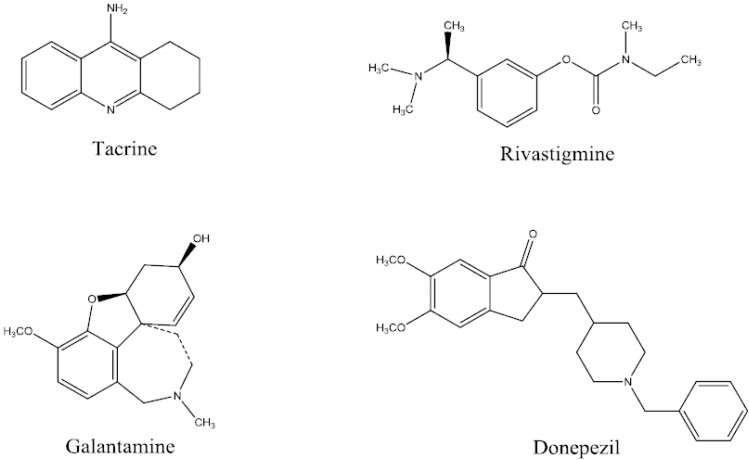
Synthetic pathway for synthesis of compounds 4a-4h

**Table 1:** Physicochemical properties of compounds 3 and 4a-4h

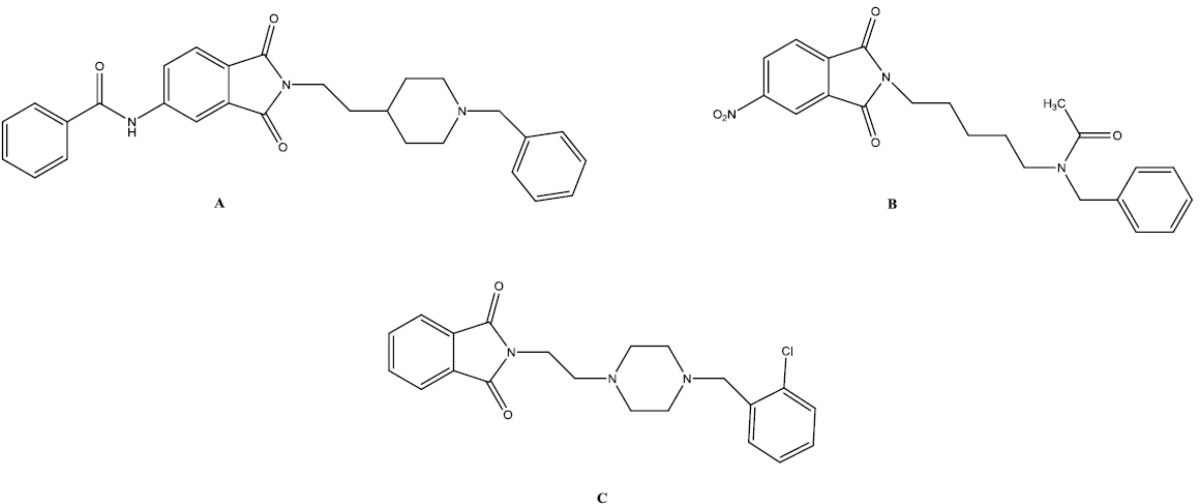

**Table 2:** Results of enzymatic assay (IC_50_, µM) of compounds 4a-4h

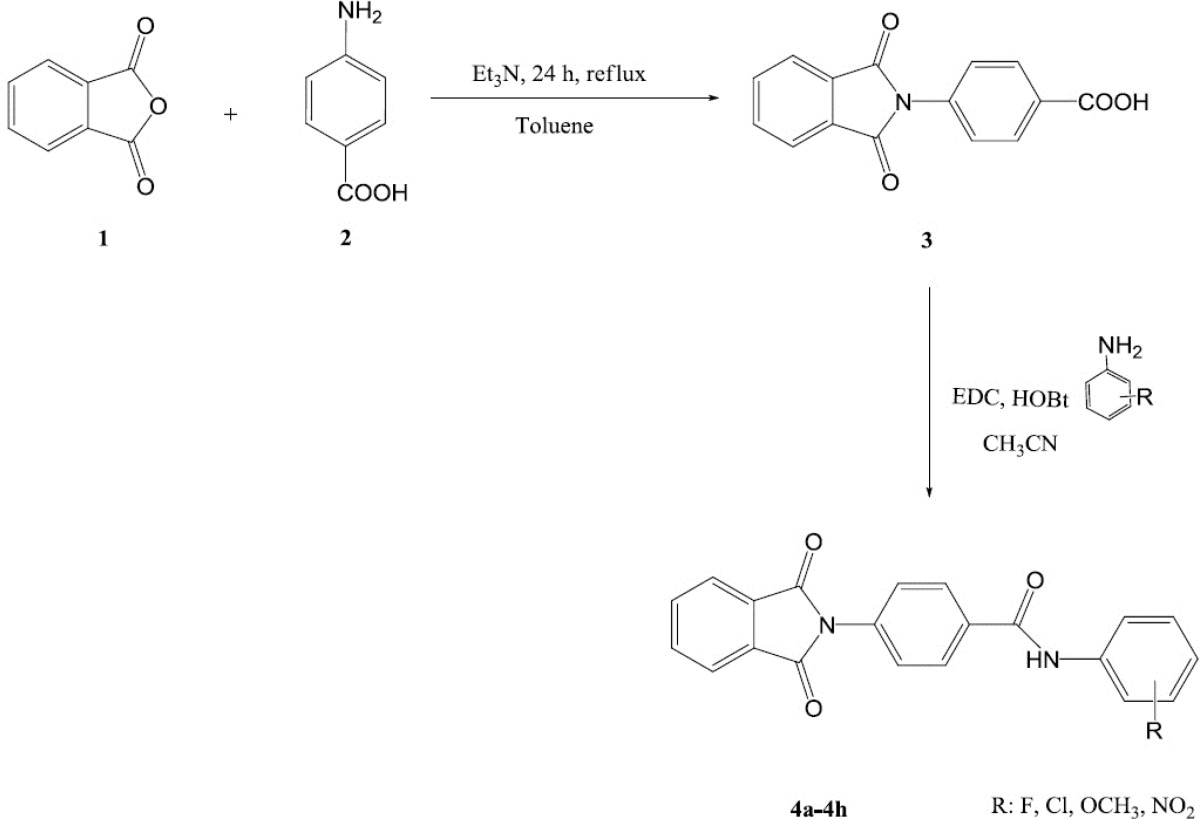

## Experimental

*Chemistry*

All starting materials, reagents and solvents were purchased from commercial suppliers like Merck and Aldrich companies. The purity of the prepared compounds was proved by thin layer chromatography (TLC) using various solvents of different polarities. Merck silica gel 60 F_254_ plates were applied for analytical TLC. Column chromatography was performed on Merck silica gel (70-230 mesh) for purification of intermediate and final compounds. ^1^H-NMR spectra were recorded using a Bruker 400 MHz spectrometer, and chemical shifts are expressed as δ (ppm) with tetramethylsilane (TMS) as internal standard. The IR spectra were obtained on a Shimadzu 470 spectrophotometer (potassium bromide disks). Melting points were determined using electrothermal melting point analyzer apparatus and are uncorrected. The mass spectra were run on a Finigan TSQ-70 spectrometer (Finigan, USA) at 70 eV.

*Synthesis of 4-(1,3-Dioxoisoindolin-2-yl)benzoic acid *(3):

In a flask 3 g (20 mmol) of Phthalic anhydride, 2.78 g (20 mmol) 4-aminobenzoic acid and 2.9 mL (20 mmol) triethylamine (Et_3_N) were mixed in 50 mL of toluene solvent (Scheme 1). The reaction mixture was refluxed for 24 h and the termination of reaction and formation of the desired product was confirmed by thin layer chromatography. The discoloration of the reaction medium and formation of a yellow precipitate was also an indicator of the progress of the reaction. Then, toluene was evaporated under reduced pressure using rotary evaporator apparatus and the obtained white powder was washed several times by *n*-hexane and diethyl ether (Et_2_O) (15-17).

^1^HNMR (DMSO-d_6_, 250 MHz) δ: 7.60 (d, 2H, *J* = 10 Hz, H_3,5_-Phenyl), 7.94 (m, H_5,6_-Phthalimide), 7.98 (m, H_4,7_-Phthalimide), 8.09 (d, 2H, *J* = 10 Hz, H_2,6_-Phenyl). IR (KBr, cm^-1^) ῡ: 3483 (N-H, Stretch, Amide), 3020 (C-H, Stretch, Aromatic), 1712 (C=O, Stretch, Phthalimide), 1689 (C=O, Stretch, Acid), 1608 (C=C, Stretch, Aromatic), 1581, 1516, 1427 (C=C, Stretch, Aromatic), 1381, 1292, 1226, 1176, 1118, 1083, 925, 891, 856, 794, 771, 713, 551, 532, 509. 

*General procedure for synthesis of compounds *4a-4h:

According to the scheme 1, in a flat bottom flask compound 3 was mixed with equimolar quantities of *N*-ethyl-*N*-dimethyl aminopropyl carbodiimide (EDC) and hydroxyl benzotriazole (HOBt) in acetonitrile solvent and was stirred for 30 min at room temperature. Then, equimolar quantity of appropriate aniline derivative was added to the reaction medium. The stirring was continued for 24 h. After confirmation of the reaction end by TLC, the acetonitrile was evaporated and water/ethyl acetate mixture was added to the residue. The organic phase was separated and was washed two times by sodium bicarbonate 5%, diluted sulfuric acid and brine. Anhydrous sodium sulfate was added, filtered and the ethyl acetate was evaporated. The obtained powder was washed by diethyl ether, *n*-hexane and purified by column chromatography (EtOAc/Petroleum ether) (18-21).

*4-(1,3-Dioxoisoindolin-2-yl)-N-(2-fluorophenyl)benzamide* (4a):

^1^HNMR (DMSO-d_6_, 250 MHz) δ: 7.32 (m, 1H, 2-Fluorophenyl), 7.62 (d, 2H, *J* = 10 Hz, Phenyl), 7.68 (m, 1H, 2-Fluorophenyl), 7.94 (m, 2H, H_5,6_-Phthalimide), 7.99 (m, 2H, H_4,7_-Phthalimide), 8.29 (m, 4H, Aromatic), 10.25 (brs, NH). IR (KBr, cm^-1^) ῡ: 3410 (N-H, Stretch, Amide), 3070 (C-H, Aromatic), 1712 (C=O, Phthalimide), 1658 (C=O, Stretch, Amide), 1604 (C=C, Stretch, Aromatic), 1508 (N-H, Bend), 1381 (C-F, Stretch). 

*4-(1,3-Dioxoisoindolin-2-yl)-N-(3-fluorophenyl)benzamide* (4b):

^1^HNMR (DMSO-d_6_, 250 MHz) δ: 7.36 (m, 6H, Aromatic), 7.95 (m, H_5,6_-Phthalimide), 7.99 (m, H_4,7_-Phthalimide), 8.08 (d, 2H, *J* = 10 Hz, H_2,6_-Phenyl), 10.54 (brs, NH). IR (KBr, cm^-1^) ῡ: 3394 (N-H, Stretch, Amide), 1716 (C=O, Phthalimide), 1658 (C=O, Stretch, Amide), 1604 (C=C, Stretch, Aromatic), 1438 (C=C, Stretch, Aromatic), 1384 (C-F, Stretch). MS (*m/z*, %): 360 (M^+^, 60), 267 (15), 250 (100), 222 (80), 194 (15), 166 (55), 139 (25), 104 (45), 90 (15), 83 (12), 76 (50), 63 (15), 50 (15). 

*N-(3-Chlorophenyl)-4-(1,3-dioxoisoindolin-2-yl)benzamide* (4c):

^1^HNMR (DMSO-d_6_, 250 MHz) δ: 7.17 (d, 1H, *J* = 7.5 Hz, H_6_-3-Chlorophenyl), 7.36 (t, 1H, *J* = 7.5 Hz, H_5_-3-Chlorophenyl), 7.63 (d, 1H, *J* = 7.5 Hz, H_3,5_-Phenyl), 7.72 (d, 1H, *J* = 7.5 Hz, H_4_-3-Chlorophenyl), 7.93 (m, 2H, H_5,6_-Phthalimide), 7.95 (m, 2H, H_4,7_-Phthalimide), 7.96 (s, 1H, H_2_-3-Chlorophenyl), 8.07 (d, 1H, *J* = 7.5 Hz, H_2,6_-Phenyl), 10.50 (brs, NH). IR (KBr, cm^-1^) ῡ: 3448 (N-H, Stretch, Amide), 1712 (C=O, Stretch, Phthalimide), 1654 (C=O, Stretch, Amide), 1593 (C=C, Stretch, Aromatic), 1504 (N-H, Bend), 1481 (C=C, Stretch, Aromatic). MS (*m/z*, %): 376 (M^+^, 2), 250 (25), 222 (15), 166 (30), 146 (15), 126 (30), 99 (90), 90 (70), 76 (100), 63 (45), 50 (30). 

*N-(4-Chlorophenyl)-4-(1,3-dioxoisoindolin-2-yl)benzamide* (4d):

^1^HNMR (DMSO-d_6_, 250 MHz) δ: 7.37 (d, 2H, *J* = 7.5 Hz, H_2,6_-4-Chlorophenyl), 7.58 (d, 2H, *J* = 7.5 Hz, H_3,5_-Phenyl), 7.82 (d, 2H, *J* = 7.5 Hz, H_3,5_-4-Chlorophenyl), 7.93 (m, 2H, H_5,6_-Phthalimide), 7.95 (d, 2H, *J* = 7.5 Hz, H_2,6_-Phenyl), 7.98 (m, 2H, H_4,7_-Phthalimide), 10.47 (brs, NH). IR (KBr, cm^-1^) ῡ: 3425 (N-H, Stretch, Amide), 1716 (C=O, Stretch, Phthalimide), 1654 (C=O, Stretch, Amide), 1627 (C=C, Stretch, Aromatic), 1519 (N-H, Bend), 1469 (C=C, Stretch, Aromatic).

*4-(1,3-Dioxoisoindolin-2-yl)-N-(2-nitrophenyl)benzamide* (4e):

^1^HNMR (DMSO-d_6_, 250 MHz) δ: 6.60 (t, 1H, *J* = 7.5 Hz, H_4_-2-Nitrophenyl), 7.00 (t, 1H, *J* = 7.5 Hz, H_6_-2-Nitrophenyl), 7.39 (m, 8H, H_3,5_-Phenyl, H_3,5_-2-Nitrophenyl, Phthalimide), 7.98 (d, 2H, H_2,6_-Phenyl), 10.45 (brs, NH). IR (KBr, cm^-1^) ῡ: 3444 (N-H, Stretch, Amide), 1712 (C=O, Stretch, Phthalimide), 1627 (C=O, Stretch, Amide), 1570 (N-H, Bend, Amide), 1504 (Stretch, Asymmetric, NO_2_), 1435 (C=C, Stretch, Aromatic), 1346 (Stretch, Symmetric, NO_2_), 1257 (C-N, Stretch). MS (*m/z*, %): 387 (M^+^, 5), 368 (10), 316 (12), 295 (12), 281 (30), 267 (65), 257 (15), 250 (100), 241 (15), 236 (25), 225 (15), 224 (70), 222 (90), 211 (20), 206 (20).

*4-(1,3-Dioxoisoindolin-2-yl)-N-(4-nitrophenyl)benzamide* (4f):

^1^HNMR (DMSO-d_6_, 250 MHz) δ: 6.56 (m, 4H, aromatic), 6.71 (brs, 4H, Phthalimide), 7.94 (m, 4H, aromatic), 10.48 (brs, NH). IR (KBr, cm^-1^) ῡ: 3363 (N-H, Stretch, Amide), 1712 (C=O, Stretch, Phthalimide), 1631 (C=O, Stretch, Amide), 1593 (C=C, Stretch, Aromatic), 1473 (C=C, Stretch, Aromatic), 1303 (C-N, Stretch).

*4-(1,3-Dioxoisoindolin-2-yl)-N-(3-methoxyphenyl)benzamide* (4g):

^1^HNMR (DMSO-d_6_, 250 MHz) δ: 3.78 (s, 3H, -OCH_3_), 6.71 (d, 1H, *J* = 10 Hz, H_6_-3-Methoxyphenyl), 7.27 (t, 1H, *J* = 7.5 Hz, H_5_-3-Methoxyphenyl), 7.40 (d, 1H, *J* = 10 Hz, H_4_-3-Methoxyphenyl), 7.50 (s, 1H, H_2_-3-Methoxyphenyl), 7.64 (d, 2H, *J* = 10 Hz, H_2,6_-Phenyl), 7.94 (m, 2H, H_5,6_-Phthalimide), 8.00 (m, 2H, H_4,7_-Phthalimide), 8.07 (d, 2H, *J* = 10 Hz, H_2,6_-Phenyl), 10.33 (brs, NH). IR (KBr, cm^-1^) ῡ: 3387 (N-H, Stretch, Amide), 2924 (C-H, Asymmetric, Aliphatic), 2854 (C-H, Symmetric, Aliphatic), 1712 (C=O, Phthalimide), 1658 (C=O, Stretch, Amide), 1600 (C=C, Stretch, Aromatic), 1527 (N-H, Bend), 1431 (C=C, Stretch, Aromatic), 1373, 1273 (C-O, Stretch, Methoxy), 1049, 844. MS (*m/z*, %): 372 (M^+^, 80), 250 (100), 222 (40), 194 (10), 166 (30), 139 (12), 104 (20), 90 (10), 76 (20), 63 (5), 50 (5).

*4-(1,3-Dioxoisoindolin-2-yl)-N-(4-methoxyphenyl)benzamide* (4h):

^1^HNMR (DMSO-d_6_, 250 MHz) δ: 3.76 (s, 3H, -OCH_3_), 6.95 (d, 1H, *J* = 10 Hz, H_3,5_-4-Methoxyphenyl), 7.62 (d, 2H, *J* = 10 Hz, H_3,5_-Phenyl), 7.70 (d, 2H, *J* = 10 Hz, H_2,6_-4-Methoxyphenyl), 7.94 (m, 2H, Phthalimide), 8.01 (m, 2H, Phthalimide), 8.07 (d, 2H, *J* = 10 Hz, H_2,6_-Phenyl), 10.24 (brs, NH). IR (KBr, cm^-1^) ῡ: 3425 (N-H, Stretch, Aromatic), 2924 (C-H, Asymmetric, Aliphatic), 2858 (C-H, Symmetric, Aliphatic), 1712 (C=O, Stretch, Phthalimide), 1651 (C=O, Stretch, Amide), 1631, 1600 (C=C, Stretch, Aromatic), 1519 (N-H, Bend, Amide), 1469 (C=C, Stretch, Aromatic), 1238 (C-N, Stretch).

*Anti-acetylcholinesterase assay*

Lyophilized powder of acetylcholinesterase from electric eel source (AChE, E.C. 3.1.1.7, Type V-S, 1000 unit) was purchased from Sigma-Aldrich (Steinheim, Germany). 5,5′-Dithiobis-(2-nitrobenzoic acid, DTNB), potassium dihydrogen phosphate (KH_2_PO_4_), dipotassium hydrogen phosphate (K_2_HPO_4_), potassium hydroxide (KOH), sodium hydrogen carbonate (NaHCO_3_), and acetylthiocholine iodide were purchased from Fluka (Buchs, Switzerland). Spectrophotometric measurements were run on a Cecil BioAquarius CE 7250 Double Beam Spectrophotometer.

Compounds 4a-4h were dissolved in a mixture of 20 mL distilled water and 5 mL methanol and then diluted in 0.1 M KH_2_PO_4_/K_2_HPO_4_ buffer (pH 8.0) to yield a final concentration range. According to the literature, the Ellman test was performed for assessment of the anticholinesterase activity of intended compounds *in-vitro*. To achieve 20-80% inhibition of AChE activity five different concentrations of each compound were tested. Compounds 4a-4h were added to the assay solution and preincubated at 25 °C with the enzyme for 15 min followed by adding 0.075 M of acetylthiocholine iodide. After rapid and immediate mixing the change of absorption was measured at 412 nm. 

The blank reading contained 3 mL buffer, 200 µL water, 100 µL DTNB and 20 µL substrate. The reaction rates were calculated, and the percent inhibition of test compounds was determined. Each concentration was analyzed in triplicate, and IC_50_ ± SD values were determined graphically from inhibition curves (log inhibitor concentration vs. percent of inhibition) (22, 23).

## Results and Discussion

*Chemistry*

All final compounds 4a-4h were synthesized according to the Scheme 1. Starting from phthalimide and 4-aminobenzoic acid through a Gabriel like reaction the intermediate compound 3 were prepared. Triethylamine (Et_3_N) was added to the reaction medium as a proton acceptor. The reaction was done under reflux condition in toluene solvent for 24 h. Thin layer chromatography (TLC) was applied for determining the end of the reaction. *n*-Hexane and diethyl ether (Et_2_O) was utilized for trituration of the obtained precipitate. The obtained white precipitate of compound 3 was used for the next reaction without any extra purification. *N*-Ethyl-*N*-dimethylaminopropyl carbodiimide (EDC) as carbodiimide coupling agent and hydroxybenzotriazole (HOBt) as additive agent were used for preparation of the final amidic derivatives 4a-4h. ^1^H NMR spectroscopy was done in deutrated dimethylsulfoxide (DMSO-d_6_) solvent and trimethylsilane (TMS) was considered as internal standard. Potassium bromide (KBr) disk was prepared for all compounds for acquisition of the IR spectra. According to Table 1, all compounds were synthesized with an average yield. Compound 4c with *ortho* fluorine moiety showed the lowest yield 37% whereas, compound 4g with *ortho* nitro substituent demonstrated the highest yield 69%. Melting point analyzer apparatus was applied for measuring the corresponding melting point of all prepared compounds.

*Anticholinesterase activity*

Ellman᾽s test was performed for evaluating the anticholinesterase activity of compounds 4a-4h. Acetylcholinesterase enzyme extracted from electric eel was utilized for assessment of the potency of intended compounds. Inhibitory concentration (IC_50_, µM) was calculated and listed in Table 2. Unfortunately, none of the tested derivatives does not exerted stronger potency than donepezil as reference drug. Only compound 4g with *ortho* nitro moiety rendered the highest inhibitory potency (IC_50_ = 1.1 ± 0.25 µM) compared to donepezil (IC_50_ = 0.41 ± 0.12) µM. Nitro moiety is an electron withdrawing group. But, this moiety caused a significant enhancement in enzyme inhibitory effect at position *ortho* of the phenyl ring. Whereas, it was not showed any remarkable potency at other positions of the phenyl ring and also replacement of this substituent with other electron withdrawing moiety such as chlorine and fluorine does not rendered any favorable improvement in inhibitory activity towards the enzyme. It is likely that electrostatic charge property and interaction of the nitro moiety maybe a rational reason for the deep difference in influencing the activity compared to other moieties. On the other hand, it is probable that positioning of the nitro group is also another critical factor for interaction to the receptor. Namely, *ortho* position (compound 4g) is better than *para* position (compound 4h) for exerting the electrostatic interaction of this moiety. 

*The authors have been declared no conflict of interest.*
